# Malagasy Amphibian Wildlife Trade Revisited: Improving Management Knowledge of the Trade

**DOI:** 10.3390/ani13142324

**Published:** 2023-07-17

**Authors:** Angus I. Carpenter, Franco Andreone

**Affiliations:** 1 Institute of Science and Environment, University of Cumbria, Ambleside Campus, Rydal Road, Ambleside, Cumbria LA22 9BB, UK; 2 Museo Regionale di Scienze Naturali, Via G. Giolitti, 36, I-10123 Torino, Italy; franco.andreone@rehione.piemonte.it

**Keywords:** wildlife trade, Madagascar, amphibians, CITES, IUCN Redlist, conservation

## Abstract

**Simple Summary:**

There is much debate about the wildlife trade, with arguments made both for and against the trade. If wildlife trade is to continue, both knowledge of species and their population statuses and confidence within the global mechanism that monitors and manages the wildlife trade are required. Using Madagascar’s amphibian trade, this study investigates this issue. The findings of the study highlight the need to maintain awareness of changes to species descriptions and the need to cross-referencing with population status data, such as that available from the IUCN Redlist, but significantly against CITES quotas and the quality of the NDFs that support them. In this study, it was observed that Madagascar seems to have improved its management of the amphibian trade over time but that new species are constantly being described over time, which could add complications to the management of the trade.

**Abstract:**

Madagascar is a biodiversity hotspot with a long history of trading in its wildlife, especially its hyper-diverse amphibian taxa. Due to globally raised concerns over the conservation of harvested species, CITES was introduced as a global mechanism with which to monitor and regulate the trade. Utilising data collated from the CITES Trade database, this study sought to investigate the trade and CITES’ effectiveness in managing the trade with respect to Madagascar. Over a 28-year period, 20 known amphibian species were exported from Madagascar, constituting a total of nearly 271,000 individuals. Formal descriptions of Malagasy amphibian species have increased and continue to increase greatly over time. However, there was no longitudinal relationship regarding the numbers of individuals traded as new species were described. Overall, the number of individuals traded has declined over time, but where assessments were provided by the IUCN Redlist, population declines were reported in all but one species of Malagasy amphibian. *Mantella* (97.5%) continues to be the predominantly traded genus, with certain, high-conservation-concern, species continuing to be traded. Despite initial concerns over the effectiveness of CITES’s actions, after concerted efforts, it appears that CITES’ actions were having positive impacts on regulating the trade. However, going forward, concerns remain over the appropriateness of the quotas set and the robustness of their underpinning NDFs. Furthermore, with the increase in the number of recognised species, the potential for incorrect species labelling on the CITES permits increases and requires greater attention.

## 1. Introduction

Academics may debate the existence of the Anthropocene [1], but no such debate rages around the extinction crisis currently underway globally [2]. Certain taxa have been reported as being more exposed to extinction risk than others due to a variety of pressures [1,3]. One such taxon is the Amphibians, having experienced high extinction rates globally due to a range of factors [4,5], such as climate and habitat change [6], chytrids [7], and overharvesting [8], to name a few.

Madagascar has been identified as a biodiversity hotspot [9], especially due to its diverse population of amphibians, with more than 365 recorded species to date but with many more species yet to be formally described [10]. The reported conservation pressures affecting amphibians in Madagascar include habitat change [11,12], diseases [13,14], alien species [12,15,16], and the wildlife trade, with the final pressure supplying bushmeat [8] and serving the demand in the pet trade [8,17,18,19]. However, many of the studies investigating the levels of wildlife trade regarding amphibians destined for international pet markets were conducted over 10–15 years ago. For example, in 1994, just 1 *Mantella* species was traded, but this number jumped to 14 known species in 2002/2003, while between 1994 and 2003, in just the *Mantella* genus, 233,893 individuals were reported to have been traded [20,21]. However, using CITES import data, for the period from 1994 to 2006, a total of 162,000 individuals were reported to have been traded across 18 species, while Malagasy government data reported the trade of over 221,000 individuals across 91 species between 2000 to 2006 [20,21]. In a global review of amphibians, it was determined that between 1978 and 2007, the genus *Mantella* was the most heavily traded genus, with 193,600 individuals traded over 14 species, accounting for 40% of the global trade figure, while 999 individuals belonging to 1 *Dyscophus* species and 2239 individuals belonging to *Scaphiophryne gottlebei* were the only other Malagasy species on the list [8]. More recently, between 2007 and 2018, the number of reported exports was reported to amount to 71,050 individual amphibians, with the genus *Mantella* accounting for nearly 97% of the trade, followed by *Scaphiophryne* (3%) and *Dyscophus* (0.5%), while the top three traded species were *M. betsileo* (*n* = 22,737; 33%), *M. baroni* (31%), and *M. nigricans* (11%) [21]. However, whilst the values reported were appropriate at the time, the wildlife trade has since been recognised as highly dynamic in nature. For example, it has been reported that the demand in species is driven by multiple factors [22], such as what was fashionable at a given time [23,24], dynamic changes in trade networks, and both financial and actor-level participant involvement, which can all impact trading [17,19,20,21,25]. Furthermore, national or international legislation changes also can affect trading [18,20,26].

Legislation change may be globally applicable, such as the Convention on International Trade in Endangered Species of Wild Fauna and Flora (CITES), or have a regional influence, such as the European Union (EU). For example, within the EU, conservation legislation sets and dictates the policies member states must implement and act on. Regarding trade in wildlife, Council Regulation (EC) No 338/97 of 9 December 1996 on the protection of species of wild fauna and flora regulates trade, specifically the procedures within Article 189c and Article 4(6) [27]. These refer to ‘Introduction into the Community’, with Article 4(6) stating: “In consultation with the countries of origin concerned, in accordance with the procedure laid down in Article 18 and taking account of any opinion from the Scientific Review Group, the Commission may establish general restrictions, or restrictions relating to certain countries of origin, on the introduction into the Community:” [27]. Annex A of Article 4 lists species, which can change according to the information available, that were allowed or restricted entry into the EU as directed in the documentation. For example, *M. aurantiaca* was listed under Article 4(6) code ‘d’ (which stated “of live specimens of species for which it has been established that their introduction into the natural environment of the Community presents an ecological threat to wild species of fauna and flora indigenous to the Community”) but have since been assigned to code ‘b’ (“on the basis of the conditions referred to in paragraph 1(e) or paragraph 2(a), of specimens of species listed in Annex B”) [28]. Such changes in coding highlight the highly dynamic conditions within which the wildlife trade operates, especially in Madagascar.

Many factors have changed since the previous studies on the Malagasy amphibian trade were conducted. For example, political leadership has changed, while greater and more coordinated strategic conservation efforts have been made [29,30,31]. The latter actions were supported through multi-national environmental agreements (MEAs), such as CITES, with the majority of traded Malagasy species listed within CITES Appendices, with varying conditions applying to them [32,33]. While the reported numbers of the Malagasy amphibians traded has increased, so too has the number of described species, increasing from 133 species in the 1990s to 244 around 2010, 292 in 2014, and 365 in 2022 [10]. This expansion in described species affects both the number and levels of trade being reported whilst also impacting the management of the trade in various ways, such as via misidentification opportunities, taxonomic reclassifications, etc. Furthermore, the pet trade has been recognised as and reported to be a fickle trade in terms of the species in demand [22,23,24]. A further consideration that needs careful addressal is the open and dynamic nature of the CITES datasets, which, while often used for trade reviews, contain inherent relevant issues [25]. Therefore, a sufficient knowledge base is required when analysing and interpreting these data [25] in order to assess the efficacy of CITES management and facilitate robust evidence-based approaches to either adapting, removing, or increasing the conservation efforts applied to species.

Therefore, this study aims to provide the most up-to-date and comprehensive review of the global trade in CITES-listed amphibian species exported from Madagascar. This information will establish a platform of knowledge from which appropriate conservation actions can be developed and implemented to improve Malagasy amphibian conversation. This study seeks to identify the countries involved and the types, levels, and complexities of the international trade in amphibians. Specifically, we aim to answer the following questions: (1) What are the levels, dynamics, and trends in the trade? (2) Which species feature significantly in the trade conducted, and what is their conservation status? (3) What is the effectiveness of CITES’s actions regarding the trade?

## 2. Materials and Methods

The Convention on International Trade in Endangered Species of Wild Flora and Fauna (CITES) was established to facilitate the monitoring of trade in wildlife-based resources with the aim of securing the species’ future through sustainable trade (www.CITES.org (accessed on 14 May 2022)). Nation states, registered as party members, submit yearly trade reports that provide details of both imports and exports conducted within the year to CITES. These data were then collated and stored on a trade database, which is maintained by United Nations Environment Program—World Conservation Monitoring Centre (UNEP-WCMC) in Cambridge, UK (https://trade.cites.org/ (accessed on 25 February 2023)), on behalf of the CITES Secretariat [25].

Data on trade conducted between 1975 to 2022 were collated and downloaded from the CITES database on 25 February 2023. The search criteria and terms used in the collation of these data have been presented in Table 1 and cover all CITES-listed species of Malagasy amphibians. Due to the well-reported permutations, vagaries, and lack of congruence between the CITES ‘export reported’ and ‘import reported’ trade values, which has often been overlooked in several studies reporting on wildlife trade, only the reported import trade data set was utilised in the following analyses [8,25,32]. CITES quota data and status for each species were extracted from Species+ (https://www.speciesplus.net/species; accessed on 5 March 2023), while their IUCN Redlist statuses were extracted from the IUCN Redlist (https://www.iucnredlist.org/; accessed on 5 March 2023).

Variables were investigated using non-parametric tests, such as Spearman rho correlation to analyse significant relationships between variables and the Mann–Whitney U test to determine any significant differences between variables.

## 3. Results

The data collated regarding the levels of trade between 1975 and 2022 recorded the first trading event in 1994, and the last datapoint reported was in 2021, resulting in a data period covering 28 years. During this period, a total of 20 known Malagasy amphibian species, plus two unknown listings (recorded as ‘genus spp.’), and a total of 270,963 individual amphibians were reported to have been exported from Madagascar (Table 2).

**Figure 1:**
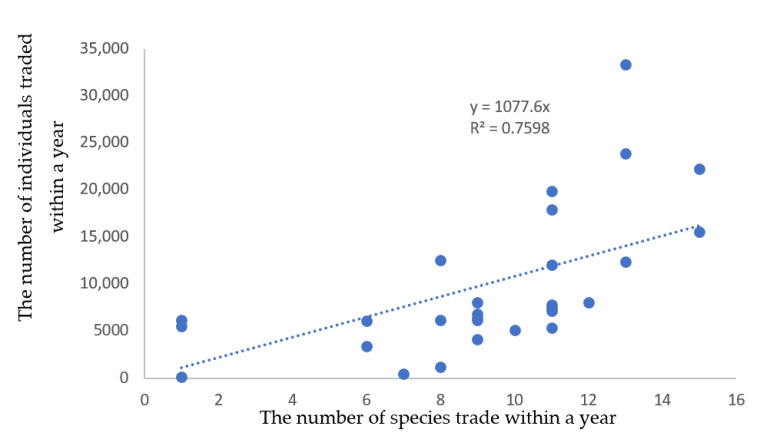
The relationship between the number of amphibian species traded and the total number of individuals traded on a yearly basis from Madagascar between 1994 and 2021 (Source: UNEP/WCMC, 2023).

**Figure 2:**
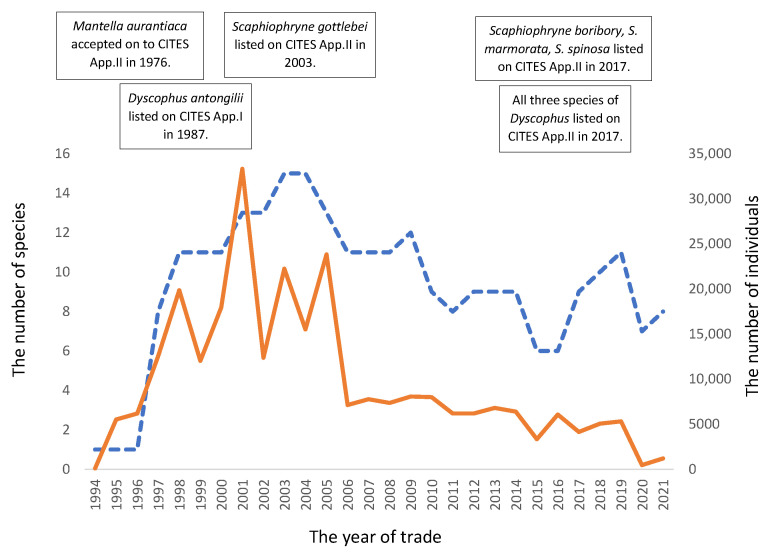
The non-linear increase in the number of species (blue dashed line and primary ‘y’ axis) exported from Madagascar each year and the total number of individuals (orange line and 2nd ‘y’ axis) reported to have been exported between 1994 and 2021 (Source: UNEP/WCMC, 2023).

Upon comparing the yearly data regarding the number of species traded with the number of individuals traded, a significant, positive relationship was observed (Figure 1; *n* = 27, *r*_s_ = 0.76481, and *p* = 0.00). However, the number of species being traded year after year did not display any longitudinal linear increase (Figure 2). Rather, after 1996, there was a rapid rise that peaked at 15 species in 2003/4 before reducing to an average of approx. 9 species traded per year between 2005 and 2021 (Figure 2). Conversely, the yearly average number of individuals traded was 9677 over the 28-year period and 6668 between 2005 and 2021, with a peak of 33,313 individuals in 2001 (Figure 2).

All 20 known species traded (Table 3) were listed on CITES App.II, while 1 (5%) was categorised as Critically Endangered (‘CR’) as per the IUCN Redlist, 6 (30%) were Endangered (‘EN’), 4 (20%) were Vulnerable (‘VU’), 1 (5%) was Near Threatened (‘NT’), and 8 (40%) were of Least Concern (‘LC’) (Table 3). Grouping the species into their IUCN Redlist categorises revealed that the greatest level of trade was in the Least Concern species (8 spp.; 95,902 individuals) closely followed by Endangered (6 spp.; 92,634 individuals), Vulnerable (4 spp.; 34,849 individuals), Near Threatened (1 spp.; 21,147 individuals), and Critically Endangered (1 spp.; 6,043 individuals) species. However, analysing trade proportionally, the category listings altered, with the Near-Threatened (21,147 individuals per spp.) species being the most traded, followed by Endangered (15,439 individuals per species), Least Concern (11,987 individuals per species), Vulnerable (8712 individuals per species), and Critically Endangered (6043 individuals per species) species. Furthermore, over 71% (179,763 individuals) of the trade was conducted in species with reported declining populations (Table 3).

**Figure 3:**
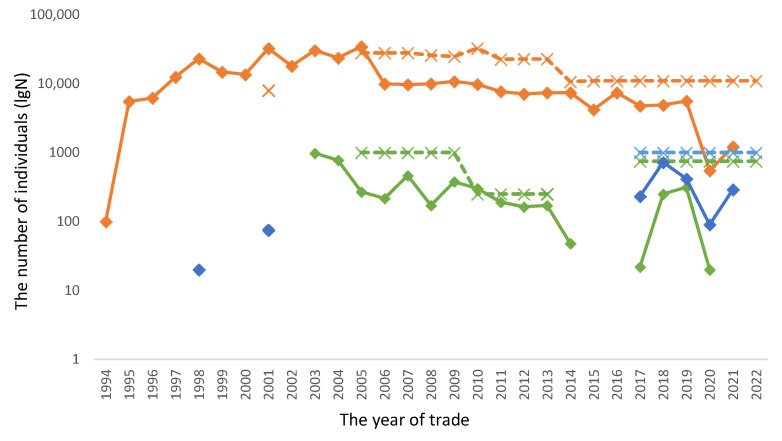
The relationship between the actual number of individuals exported from Madagascar for each of the three amphibian genera (*Mantella*, *Scaphiophryne*, and *Dyscophus*) and the CITES quotas implemented each year as reported between 1994 and 2021 (Source: UNEP/WCMC, 2023). Brown represents *Mantella*; green represents *Scaphiophryne*, and blue represents *Dyscophus*, while solid lines with diamonds denote reported export numbers, and dashed lines with open crosses denote the CITES quota levels.

To manage resources, CITES utilises a quota system to limit the quantity of a particular resource. In terms of managing trade, a quota is successful when the actual trade value does not exceed the quota provided for that species by the exporting country. The relationship between actual trade conducted and the quota levels set have been presented for each genus in Figure 3. At the genus level, there were just two years when the total number of exported individuals within a genus exceeded the CITES quota total; namely, 2001 for *Mantella* (when 29,567 were exported and the quota was 8000) and 2010 for *Scaphiophryne* (when 302 were exported and the CITES quota total was 250).

**Figure 4:**
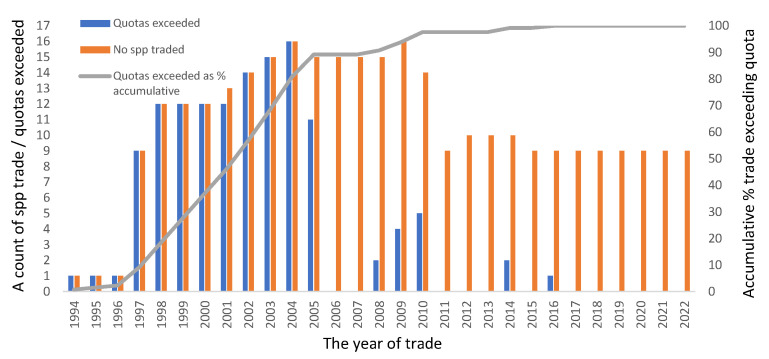
The events when reported trade numbers exceeded CITES quotas within a year as reported between 1994 and 2021 (Source: UNEP/WCMC, 2023).

**Figure 5:**
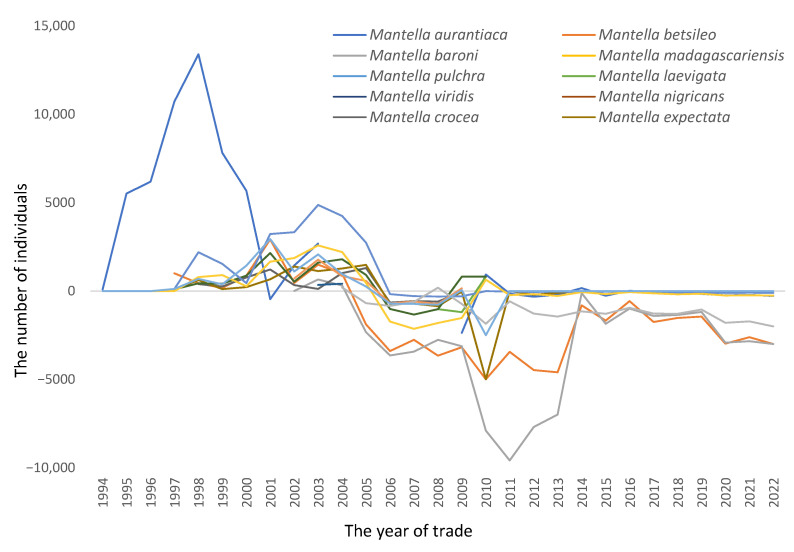
The graph shows when CITES quotas were exceeded (numbers presented > zero) or not met (numbers < zero), with the zero-reference line indicating when the reported number exported was equal to a given species’ CITES quota for data reported between 1994 and 2021 (Source: UNEP/WCMC, 2023).

However, at the species level and for each year, the reported number of amphibians exported from Madagascar exceeded the CITES quota level on many more occasions (Figure 4 and Figure 5). As *Mantella* accounted for nearly 98% of the reported total export number, this genus was focused on to explore the relationship between the reported number exported and the CITES quotas per species for each year for which comparative data existed (Figure 4 and Figure 5). The total number of occasions when the reported export number exceeded its CITES quota within a set year was 130 times over the whole period for *Mantella* species (Figure 4). Nearly 90% of these events were recorded leading up to 2005, with 16 species traded in numbers higher than their CITES quotas in 2004 (Figure 4). There were no recorded events in 2006 and 2007, while just two were recorded in 2008 with 2009 and four and five species were reported to have had their quotas exceeded in 2010; subsequently, these events reduce to nearly zero for the remainder of the period (Figure 4 and Figure 5). Where the reported number exported for a species equals the CITES quota, it is shown as zero in Figure 5, while numbers below zero correspond to species traded at levels below their CITES quotas for the corresponding year, and vice versa. The scale of departure from the CITES quota was highest in 1998 for *M. aurantiaca* (Figure 5). After an initial period of a large number of above-quota events, post-2005–2006 trade levels rarely exceeded their CITES quota but were still highly fluctuating, albeit in a negative relationship (Figure 5). Much greater consistency in the relationships between these datasets was observed post-2014 (Figure 5).

## 4. Discussion

This study aimed to investigate the trade in CITES-listed Malagasy amphibians and how effective the management of this trade was. This study has shown that the amphibians exported in the highest numbers from Madagascar to supply the international trade demand were still predominantly *Mantella* species. Of the 270,963 individual amphibians exported, over 97.5% were *Mantella* species, highlighting their continued demand in the trade, followed by *Scaphiophryne* spp. (1.7%) and *Dyscophus* spp. (nearly 0.7%). However, whilst an increasing number of *Mantella* species were recorded within the trade, especially in the post-2003/04/05 periods (with 15, 16, and 15 species exported, respectively), there was no linear increase in the yearly number of individuals reported to have been exported (Figure 1 and Figure 4). This suggests that the international trade in *Mantella* had a demand ceiling, which could be met by a few or many *Mantella* species. From a population-harvesting-impact perspective, this information suggests there was no longer a favourite *Mantella* species. Rather, the harvesting impact could be spread across many *Mantella* species and even away from species that might have experienced high levels of exploitation or other perturbations to their populations via adaptive management processes.

A similar trading pattern was observed within the *Dyscophus* genus. Prior to 2017, *Dyscophus antongilii* was listed on CITES App. I, while *D. insularis* and *D. guineti* were not listed at all. At the CITES meeting in South Africa in 2017, *D. antongilii* was downlisted from App. I to App. II, while both *D. insularis* and *D. guineti* were uplisted to CITES App. II. However, after these regulatory changes, only trade in *D. insularis* and *D. guineti* was reported, thus indicating that the pre-2017 export trade fears that had previously listed *D. antongilii* as belonging to CITES App. I had altered. It is possible that this species was no longer ‘fashionable’, that the demand had already been satiated, or that the international demand window had been usurped by ex situ, captive breeding supplying the trade, with each scenario highlighting how extremely dynamic the wildlife trade can be for these species. Therefore, this could have wider consequences for any attempts to seek local community benefits from engaging in the sustainable harvesting of species for poverty alleviation and conservation benefits.

The advances in the ex situ captive breeding of Malagasy amphibians, such as reported in other studies [8], highlight the fact that the window of opportunity for native country supply is limited, which has financial implications for conservation within these source countries. For example, in 2023, various species of captive-bred *Mantella* could be purchased for the retail price of USD 87 (GBP 70; www.reptiles.swelluk.com (accessed 11 May 2023)). The average exchange rate for the last 5 years has been 1.25, resulting in the US $87 retail price. A reported 12,046 individuals were exported from Madagascar, equating to a financial income of USD 1,048,002. It has been stated that 3% of the retail value reaches local communities [17,18], resulting in a potential loss of over USD 31k in local income in Madagascar if the native supply of such species is lost to ex situ breeders. This also excludes the income generated for intermediaries, who were also Malagasy [17,18], in addition to the Government taxes accrued and the wider contributions to businesses and jobs along the wider supply chain, such as transport.

Madagascar’s management of the trade as a potential sustainable resource was noticeable due to its apparent lack of any control on the levels of trade in amphibian species or its inability to implement CITES quotas early on (Figure 4 and Figure 5). This led to greater international attention and scrutiny in relation to Madagascar by CITES, such as the performance of country reviews. [26]. However, trading levels exceeding CITES quota levels appears to have almost ceased following 2010 (Figure 4), with just 14 events between 2010 and 2022 across a variety of *Mantella* spp. Whether this was due to increased effectiveness in the management of the trade by Madagascar, captive breeding advances in the non-native countries supplying international demands, or a combination of these and other factors requires further study. One area for future study is the appropriateness and robustness of the datasets provided by Madagascar’s government representatives to CITES that are used to calculate CITES Non-Detrimental Findings (NDFs) and serve as their basis. Upon viewing the quota values available, there seems to be a great deal of commonality in the values used despite the highly variable factors influencing each species. Thus, much greater attention needs to be paid toward increasing the robustness of the datasets that are used to calculate NDFs.

Furthermore, regarding the management of the trade and conservation statuses, as indicated by the ‘IUCN Red List of Threatened Species’ categorisation, there appears to be a lack of alignment or synchronisation between the two sectors. For example, one (5%) species was Critically Endangered (‘CR’), six (30%) were Endangered (‘EN’), and four (20%) were Vulnerable (‘VU’) under the IUCN Red List categorisation, yet they appeared to be highly traded (Table 3). However, at a finer scale, there were variabilities; for example, *Mantella milotympanum*, an IUCN Red List CR species, has not been recorded to have been exported since 2010. Conversely, *Mantella aurantiaca* (EN) has been reportedly exported every year from Madagascar over the 28-year period, except for 2004 to 2008, when no trade was recorded. Almost all the amphibian species were stated to have declining populations under the IUCN Red List; however, CITES quotas were set at 250, 500, or 3000, apparently without regard to the population trend. This highlights further possible research areas for the future, such as information sharing and synergies between CITES Non-Detrimental Findings (NDFs) and IUCN Red List categorisation. It is still unclear whether the most up-to-date information was being shared and utilised within each of the relevant working groups. Furthermore, it would be interesting to determine what degree of researcher collaboration transpired between the two organisations’ working groups to ensure alignment in actions that is collaborative rather than appearing independent.

Thus, this study highlights areas where the management of wildlife trade practice can be improved, such as the CITES management of the trade. However, this study also highlights several areas for future research that could initiate further improvements to the management of Malagasy wildlife resources. Furthermore, there must be greater communication and alignment between international institutions to improve the management and knock-on sensitivities of these wildlife resources to attain conservation benefits.

## 5. Conclusions

Malagasy amphibian biodiversity has increased continuously over time and continues to increase. The addition of new species into the trade brings into question the suitability of the data that support their inclusion. For example, the NDFs produced within a country by the CITES Management Authority (MA) to determine the quotas submitted to CITES must be investigated with regard to their robustness and reliability. However, CITES capacity building efforts could be, in part, the reason for the apparent improvement of the amphibian trade in Madagascar. Whilst the conservation and poverty alleviation benefits will continue to be debated, it is imperative that the areas of both species knowledge and management effectiveness are constantly being reviewed and improved. Studies such as this one allow for those involved in the management of wildlife trade and conservation on Madagascar to identify areas where potential easy victories can be achieved with active changes.

## Figures and Tables

**Table 1:** Criteria selected prior to performing the data collation for Amphibian trade within the CITES trade database (Source: UNEP-WCMC, 2023).

Database Field	Search Input
Search date	25 February 2023
Year range	1975–2022
Exporting countries	Madagascar
Importing countries	All countries
Source	Wild (W), Ranched (R), Source unknown (U)
Purpose	Commercial (T), Bred in captivity or artificially propagated (B), Botanical Garden (G), Circus and travelling exhibitions (Q), Personal (P)
Trade terms	Live (LIV), Specimens (SPE), Bodies (BOD)
Taxon	Amphibia (Amphibians)

**Table 2:** The species and number of individual Malagasy amphibians reported to have been exported from Madagascar in each year over the period from 1994 to 2021 (Source: UNEP/WCMC, 2023).

Amphibian Species	Year																											
	1994	1995	1996	1997	1998	1999	2000	2001	2002	2003	2004	2005	2006	2007	2008	2009	2010	2011	2012	2013	2014	2015	2016	2017	2018	2019	2020	2021
*Dyscophus antongilii*					20			75																				
*Dyscophus guineti*																								170	437	202	30	182
*Dyscophus insularis*																								62	286	215	60	108
*Dyscophus* spp.				45																								
*Mantella aurantiaca*	100	5515	6185	10,720	13,403	7815	5676	7545	1450	2681						135	1490	396	230	341	170	13	298	191	176	119	38	58
*Mantella baroni*									10	650	313	2670	1359	1570	2237	1872	2100	415	2302	3005	2880	1142	2003	1584	1642	1811	80	160
*Mantella bernhardi*						30	440	543	400	60	105	60					88	49	14	82	12							
*Mantella betsileo*				1000	435	175	872	2926	460	1490	995	3110	1599	2238	1340	1818	1845	3396	2366	2239	2187	1331	2423	1248	1472	1551	22	392
*Mantella cowanii*					52	150	170	434	241	500	120																	
*Mantella crocea*					395	250	763	1223	330	125	1020	2295	346	425	410	436												
*Mantella expectata*				100	624	105	220	660	1390	1125	1280	2475	272	278	145	219			45	147	11							
*Mantella haraldmeieri*								180		350	410																	
*Mantella laevigata*				100	435	415	869	2155	533	1606	1795	2910	991	665	973	808	813											
*Mantella madagascariensis*				125	2192	1535	450	3231	3325	4873	4245	3235	329	212	192	203	102	85	53	81	50	73	105	20	20	8		9
*Mantella milotympanum*									710	1780	850	1575	304	400	267	157												
*Mantella nigricans*											200	315	150	382	192	272	144	1421	721	556	853	709	1048	721	716	956	204	282
*Mantella pulchra*					784	905	270	1658	1870	2585	2205	3455	1269	868	1197	1480	1116	241	297	193	184	78	193	122	68	97		12
*Mantella* spp.				330	820	260	6779	9738	545	1366	255	200														50		
*Mantella viridis*				125	690	385	1434	2945	1110	2065	955	1260	295	269	224	299												
*Scaphiophryne gottlebei*										980	776	270	216	465	171	377	302	191	163	171	48							
*Scaphiophryne marmorata*																								22	80	93		
*Scaphiophryne spinosa*																									170	220	20	

**Table 3:** The Malagasy amphibian species recorded in the trade data, which are presented in order of the total number of individuals recorded in the trade and the species status according to CITES and the IUCN Red List.

Amphibian Species	Total Traded		CITES Listing		
		% of Trade		Population Trend	IUCN Redlist Status
*Mantella aurantiaca*	64,745	23.89	II	↓	EN
*Mantella betsileo*	38,930	14.37	II	↔	LC
*Mantella baroni*	29,805	11.00	II	?	LC
*Mantella madagascariensis*	24,753	9.14	II	↓	VU
*Mantella pulchra*	21,147	7.80	II	↓	NT
*Mantella* spp.	20,343	7.51		/	/
*Mantella laevigata*	15,068	5.56	II	↓	LC
*Mantella viridis*	12,056	4.45	II	↓	EN
*Mantella nigricans*	9842	3.63	II	↓	LC
*Mantella expectata*	9096	3.36	II	↓	EN
*Mantella crocea*	8018	2.96	II	↓	VU
*Mantella milotympanum*	6043	2.23	II	↓	CR
*Scaphiophryne gottlebei*	4130	1.52	II	↓	EN
*Mantella bernhardi*	1883	0.69	II	↓	VU
*Mantella cowanii*	1667	0.62	II	?	EN
*Dyscophus guineti*	1021	0.38	II	↓	LC
*Mantella haraldmeieri*	940	0.35	II	↓	EN
*Dyscophus insularis*	731	0.27	II	↓	LC
*Scaphiophryne spinosa*	410	0.15	II	?	LC
*Scaphiophryne marmorata*	195	0.07	II	↓	VU
*Dyscophus antongilii*	95	0.04	II	↓	LC
*Dyscophus* spp.	45	0.02		/	/

## Data Availability

CITES species trade data were openly and freely available from CITES Trade Database.
